# Development and Validation of the Dietary Habits and Colon Cancer Beliefs Survey (DHCCBS): An Instrument Assessing Health Beliefs Related to Red Meat and Green Leafy Vegetable Consumption

**DOI:** 10.1155/2019/2326808

**Published:** 2019-03-19

**Authors:** Kristen S. Smith, Savannah V. Raney, Michael W. Greene, Andrew D. Frugé

**Affiliations:** Department of Nutrition, Dietetics and Hospitality Management, Auburn University 101 Poultry Science Building, 260 Lem Morrison Drive, Auburn, AL 36849, USA

## Abstract

Dietary patterns characterized by higher red meat (RM) consumption are associated with increased colon cancer (CC) risk. Preclinical and epidemiological evidence suggest higher green leafy vegetable (GLV) consumption may mitigate these risks. Determining the relationship between dietary habits and expected health outcomes is needed.* Methods*. The Health Belief Model (HBM) was used to assess perceived CC susceptibility and severity, and related dietary benefits, barriers, and motivators. RM and GLV consumption were quantified using select DHQII items (n=15) capturing the previous 30 days' intake. A 34-item Qualtrics survey was provided to a convenience sample of 1,075 adults residing throughout the US Confirmatory factor analysis measured fitness with HBM, and Cronbach's alpha assessed subscale reliability. A subsample (n=47) completed a 2-week follow-up for test-retest reliability. Independent sample t-tests were used to compare RM and GLV intake and DHCCBS responses between genders. Individual barrier questions and RM and GLV consumption were compared using ANOVA for each gender; post hoc analyses between barrier question responses were assessed with Bonferroni correction. Results were considered significant with a p value of less than 0.05.* Results*. 990 US adults (52.7% female, 79.1% white, 50.8% aged 35+ years) completed valid surveys. Factor analysis with varimax rotation validated the construct of HBM subscales; only one question had a loading less than 0.745. Subscale Cronbach's alphas ranged within 0.478-0.845. Overall test-retest reliability was acceptable (r=0.697, p=5.22x10^−8^). Participant BMI was (mean±SD) 26.7±6.6 kg/m^2^. Participants consumed (median, IQR) 2.3, 0.9-4.7 cooked cup equivalents GLV/week and 12.2, 5.8-21.5 ounces RM/week. Over half of respondents agreed or strongly agreed with the statement “I can't imagine never eating red meat,” while less than one eighth of respondents agreed or strongly agreed with the statement “I don't like the taste of green leafy vegetables.”* Conclusion*. The DHCCBS is a valid instrument for measuring health beliefs related to red meat, green leafy vegetables, and perceived colon cancer risk. Additionally, these findings suggest increasing GLV may be more feasible than reducing RM for CC risk reduction in meat eaters.

## 1. Background

Colon cancer (CC) is the third most common cancer worldwide, and in the United States. While the incidence and mortality rate of CC have declined since 2000 due to screening, they have since risen recently in younger age groups and remain high in states with a high incidence of obesity [1]. It is the leading cause of cancer death among nonsmokers in affluent countries [2]. Lifestyle factors greatly influence CC risk, such as dietary patterns and cooking methods [3]. The Western diet, characterized by high meat and low vegetable intake, has been associated with greater risk of developing CC [4, 5]. Red meat (RM) has a greater influence on CC development than poultry, due to its high heme content, which has a catalytic effect on carcinogenic compounds, and the formation of cytotoxins within the gut [4, 6–9]. Furthermore, certain RM cooking methods, such as pan-frying and broiling, can increase exposure to carcinogenic compounds such as heterocyclic amines (HCA) and polycyclic aromatic hydrocarbons (PAH) [8]. Diets high in green leafy vegetables (GLV) are associated with reduced risk for CC [6, 10, 11]. GLV are rich in fiber, folate, and chlorophyll, contributing to their cancer-protective effects. Fiber acts as a prebiotic and can improve fecal transit time [12], folate helps to maintain gene stability [10], and chlorophyll, which binds heme in its hydrophobic phytol tail, prevents heme-induced damage in the gut epithelium [6].

Dietary patterns in the United States are typically high in RM and low in GLV or vice versa [13]. Whether meat eaters will increase GLV consumption to reduce CC risk is unknown [14]. Most individuals do not seek change unless they perceive they are vulnerable to the condition and expect benefits from preventative actions [15]. Therefore, health beliefs and attitudes toward diet and CC must be better understood in order to develop effective risk-reducing dietary interventions [14].

The Health Belief Model (HBM), developed in 1950s, has been consistently used to investigate factors influencing health behaviors [15]. The HBM measures five domains: perceived susceptibility, perceived severity, perceived benefit, perceived barriers, and cues to action. The HBM has been used to assess behaviors related to osteoporosis prevention, breast cancer screening, public awareness of cancer, reversal of metabolic syndrome, and intent to receive the H1N1 vaccine [16–22]. We developed the Dietary Habits and Colon Cancer Beliefs Survey (DHCCBS) using the HBM to explore beliefs and attitudes related to diet and CC risk. Additionally, we explored the relationships between DHCCBS, dietary intake, and demographics of survey respondents.

## 2. Methods

### 2.1. Instrument

The survey instrument consisted of 46 questions; twenty questions from the Dietary Health Questionnaire II (DHQII) measured green vegetable and red meat intake over the previous 30 days [23, 24]. Red meat questions included intake of red meat (beef, pork, and lamb) and processed meats (bacon, sausages, deli meats, etc.).Three additional previously validated Meat Module Questionnaire (MMQ) questions developed to assess exposure to HCA and PAH were added regarding the preferred meat temperatures for burgers, steak, and bacon [25]. Temperature was an alternate measure of these carcinogens in meats and determined to be most prevalent in burgers, steak, and bacon during the National Cancer Institute's development of this survey. The DHCCBS was developed to analyze health behaviors and attitudes towards CC using HBM domains. Two attention check questions (e.g., answer two times per week to this question) were included to validate survey responses. The remaining 8 questions on the survey assessed demographic and anthropometric information (gender, age, education, marital status, height, and weight).

### 2.2. Scoring of the Instrument

DHQ II answers assessing frequency of meat and vegetable intake were never, 1 time in the past month, 2-3 times in the past month, 1 time per week, 2 times per week, 3-4 times per week, 5-6 times per week, 1 time per day, and 2 or more times per day. Serving sizes were multiplied by their respective daily frequencies of consumption (0, 0.033, 0.083, 0.143, 0.285, 0.5, 0.785, 1.0, and 2.0) to estimate serving sizes. Amounts consumed were converted into cooked cup equivalents for GLV and ounce equivalents for RM and then multiplied by 7 to be reported as weekly consumption amounts.

The DHCCBS consisted of questions from the following HBM domains: one susceptibility question, two severity questions, two barrier questions, three benefits questions, and four cues to action questions. Each question was answered on a 5-point Likert scale, strongly disagree, disagree, neither agree nor disagree, agree, strongly agree, and scored as 1, 2, 3, 4, and 5, respectively.

### 2.3. Participants

Approval was granted for this study through Auburn University's Institutional Review Board. Participants were informed about confidentiality and their right to discontinue at any time prior to completing the survey. Consent was inferred after reading the IRB information letter and the survey commenced.

Participants were recruited in May 2018 through the online portal Amazon Mechanical Turk to complete the survey instrument. The survey was administered through Qualtrics, and participants were compensated $0.50 upon completion of a valid survey.

### 2.4. Statistical Analysis

The sample size for this study was determined using the widely cited rule of thumb suggesting a 1:10 variable to subject ratio [26]. Additional consideration for collecting a large enough sample from respondents in the southeastern United States led to a target sample size of 1000. For test-retest reliability, the HBM items in the retest were scored and summated as a total score. A Pearson correlation coefficient of 0.6 or greater was considered acceptable.

Instrument validity was determined through exploratory factor analysis. Any factor with an eigenvalue greater than 1.00 was considered significant. Confirmatory factor analysis evaluated factor groupings within the HBM. Varimax rotation was used to determine the extracted factors, and factor loading level was acceptable if it had a score ≥ 0.4.

Exploratory analyses were conducted to assess relationships between DHCCBS questions and RM and GLV intake between genders. Independent sample t-tests were used to compare RM and GLV intake and DHCCBS responses between genders. Individual barrier questions and RM and GLV consumption were compared using one-way analysis of variance for each gender; post hoc analyses between barrier question responses were assessed with Bonferroni correction for multiple comparisons. Results were considered significant with a p value of less than 0.05.

## 3. Results

A total of 1075 surveys were completed. After exclusions for incorrect attention check questions answers (n=67), duplicate survey attempts (n=15), and incorrect mTurk codes (n=3), 990 surveys were deemed valid. Of the 990 participants, 52.7% were female, 40.5% were single, 79.6% were white, and 54.4% had at least a Bachelor's Degree (Table 1).

Exploratory factor analysis was used to assess HBM validity. Each factor corresponded to the questions within each domain, thus validating the construct of domain questions. The loading scale scores were 0.916, 0.847, 0.662, and 0.773 for severity, benefits, barriers, and cues to action, respectively. The Cronbach's alpha coefficient scores were 0.845, 0.704, 0.478, and 0.778 for severity, benefits, barriers, and cues to action, respectively. Test re-test reliability was assessed by providing respondents the opportunity to complete the HBM questions two weeks after completion of the DHCCBS; correlation between 47 valid retests and the respondents' original responses deemed that reliability was acceptable (*r* = 0.697,* p* = 5.22x10^−8^).

Men and women consumed similar amounts of GLV, averaging half a cup per day; however, men consumed significantly more red meat (*p* = 9.0x10^−7^) (Table 2). Men reported a higher perceived risk for CC than women (Q1:* p* = 0.023). However, there were no significant gender differences in questions regarding severity (Q2:* p* = 0.837, Q3:* p* = 0.402) and benefits (Q4:* p* = 0.876, Q5:* p* = 0.579). There were no differences between preference for other protein rich foods (Q6:* p* = 0.901). Men had a greater dislike for GLV (Q7:* p* = 0.052) and greater attachment to red meat (Q8:* p* = 0.011). Men also were more likely to acknowledge receiving healthy eating cues from friends, family, and healthcare providers (Q9:* p* ≤ 0.001, Q10:* p* = 0.003, Q11:* p* = 0.068, Q12:* p* = 0.001). Perceived barrier questions had notable differences that were further explored.

**Figure 1:**
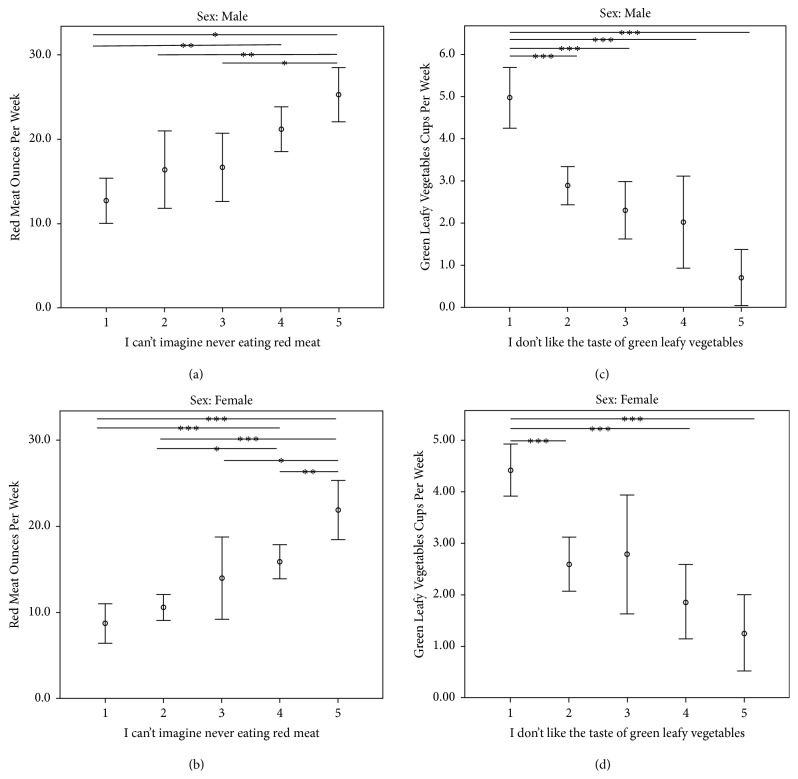
Subjective measures of red meat and green leafy vegetable intake of United States men and women relative to survey question responses regarding diet preferences. On the X-axis, the numbers 1-5 correspond to Likert scale ratings: 1 = strongly disagree; 2 = disagree; 3 = neither agree nor disagree; 4 = agree; 5 = strongly agree. Intake of food groups is reported as means with 95% confidence interval error bars. Differences in intake between response groups were assessed via ANOVA with Bonferroni correction for multiple comparisons. (a) Red meat consumption (ounces per week) of men by response categories of DHCCBS Q8: I can't imagine never eating red meat. 55.3% of men agreed or strongly agreed with Q8. (b) Red meat consumption (ounces per week) of women by response categories of DHCCBS Q8. 50.7% of women agreed or strongly agreed with Q8. (c) Green leafy vegetable consumption (cups per week) of men by response categories of DHCCBS Q7: I don't like the taste of green leafy vegetables. 14.5% of men agreed or strongly agreed with Q7. (d) Green leafy vegetable consumption (cups per week) of women by response categories of Q7. 14.9% of women agreed or strongly agreed with Q7. (∗ = p < 0.05; ∗∗ = p < 0.01; and ∗∗∗ = p < 0.005).

Given the potential of increasing GLV consumption or reducing RM intake as a CC preventive measure, we explored the relationship between two barrier questions and RM and GLV intake within each gender (Figure 1). In response to Q8: I can't imagine never eating red meat, slightly more than half of male respondents agreed or strongly agreed with the statement and consumed significantly more red meat than those who strongly disagreed (*p* < 0.001) (Table 3). In response to the same question, half of women agreed or strongly agreed and similarly consumed more RM than those who disagreed or strongly disagreed (*p* < 0.001 for both). In response to Q7: I don't like the taste of green leafy vegetables, roughly one in eight men agreed or strongly agreed; those who strongly disagreed ate significantly more GLV than all other respondents (*p* < 0.001 for all). Responses were similarly distributed in women, with those strongly disagreeing consuming more than all other categories (*p* < 0.005 for all) except those that neither agreed or disagreed (*p* = 0.232).

**Figure 2:**
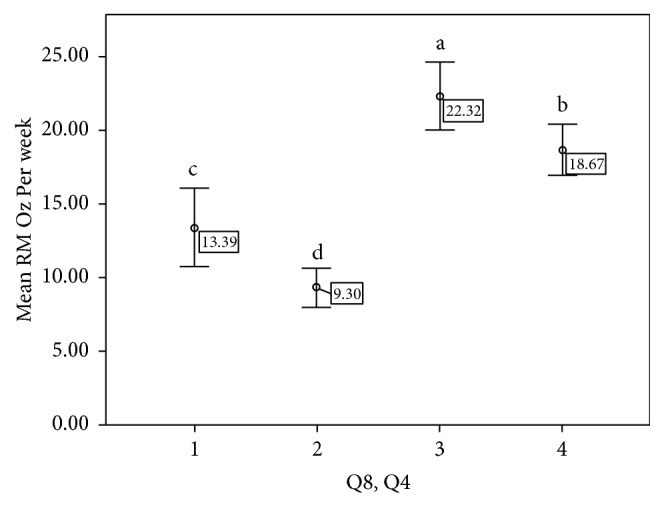
Combinations of responses to RM barrier and benefits questions compared to weekly RM intake. Q8 (I can't imagine never eating red meat) and Q4 (If I eat less red meat, I could decrease my risk for developing colon cancer). X-axis (1-4) corresponds to responses to Q8 and Q4, respectively (1 = disagree, disagree; 2 = disagree, agree; 3 = agree, disagree; 4 = agree, agree). Y-axis reports mean RM ounces per week. (1) 14.7% of individuals were non-meat lovers and did not perceive the benefits from reducing RM intake. (2) 32.3% of respondents were non-meat lovers and perceived benefits from reducing RM intake. (3) 23.4% of respondents were meat lovers and did not perceive benefits from RM reduction. (4) 29.5% of individuals were meat lovers and perceived the benefits to reduction in RM intake. Different letters indicate significant differences between groups.

We further explored the relationship between RM barriers and RM benefits relative to RM consumption by binary categorization of Q4 and Q8 responses (Figure 2). Responses were defined as either disagree (strongly disagree, disagree, and neither agree nor disagree) or agree (strongly agree and agree). There were significant differences in RM consumption across all combinations of responses (Bonferroni* p* < 0.05 was considered significant). Individuals who agreed with Q8 (meat lovers) and did not perceive benefits from less RM intake consumed approximately 8.9 ounces more than individuals who disagreed with Q8 (non-meat lovers) and did not perceive the benefits (p < 0.005). Furthermore, these meat lovers consumed over twice as much RM than non-meat lovers who perceived the benefits (p < 0.005).

## 4. Discussion

Herein, we developed and validated the new survey tool to assess dietary habits related to colon cancer beliefs and attitudes utilizing HBM to determine factors that influence actions toward colon cancer. Furthermore, we incorporated previously validated questions from DHQ II to assess habitual dietary intake, which provided further insight into health beliefs relative to subjectively measured dietary behaviors.

The HBM addresses problematic behaviors that may lead to health concerns, and has become one of the most widely used theories regarding health behaviors [15]. The six domains of the HBM are determinants of an individual's likelihood of adopting a health behavior and provide useful framework for developing effective health interventions. It has been used to provide interventions for healthy eating behaviors, as well as smoking cessation [27, 28]. Using variables from the HBM, Izadirad found significant differences from pre- to post-intervention in prenatal care and knowledge in the intervention group, but not in the control group [29]. Hazavehei discovered significant increases in knowledge about osteoporosis in middle-school-aged girls after two education intervention sessions, and this increase in knowledge was seen in perceived susceptibility, perceived severity, perceived benefits, and cues to action [30]. These studies indicate the DHCCBS may also be valuable and productive in developing effective dietary interventions for reducing CC risk.

Analysis of barrier question responses relative to consumption of RM and GLV is insightful for several reasons. First, they confirm that liking or disliking for a food is reflected in habitual consumption as measured by FFQ. Second, these findings indicate that a larger proportion of both men and women are less willing to forego RM as opposed consuming more GLV. Moreover, those who agreed or strongly agreed that they did not like GLV still consumed one or more cups on average, implying that these individuals may already be consuming GLV specifically for health benefits. Further research is needed to explore these relationships.

Internal consistency is most commonly evaluated using Cronbach's alpha coefficients. All but one factor in DHCCB survey fell within the acceptable threshold for consistency (0.70-0.95) [31]. Barrier questions (Q6-Q8) intentionally measured different obstacles, such as dislike for GLV or attachment to RM. Due to the nature of the questions, we did not expect internal consistency. These barrier questions were aimed at overlapping concepts, and examining polarizing beliefs was an effective method for exploring these attitudes.

There were limitations within the scope of this study. The generalizability of the results from this study is limited since this was not a representative sample of United States adults. Though 990 valid responses were obtained, older adults and African Americans were underrepresented, and respondents were more educated than the general population; over 50% of our respondents obtained at least a Bachelor's Degree compared to roughly one-third of Americans [32]. Findings of this survey were strengthened by dietary data, though this is an often misreported area of epidemiological research. We acknowledge that several more questions could have been included for each domain; however, given the length of the DHQII portion of the instrument, we sought to minimize respondent burden. Differences in total calories were not accounted for, as a limitation of FFQ. However, WHO guidelines are not specific to RM percentage of total calories, but rather servings of RM [33]. Furthermore, there is always risk of false disclosure or misreport of information when distributing an online survey.

The survey instrument developed and validated in the current study can be used to measure factors that influence health behaviors regarding colon cancer. Future research should examine more diverse populations in order to provide health recommendations to the general population. Based on the results of this study, DHCCBS is a valid and reliable survey instrument to assess dietary behavior related to colon cancer risk. Additionally, these findings suggest dietary guidance to increase GLV consumption may be more effective than guidance to reduce RM intake if found to be equally beneficial in reducing CC risk.

## Figures and Tables

**Table 1:** Demographic characteristics of study participants.

	Frequency (n = 990)	%
*Sex*		
Male	468	47.3
Female	522	52.7
*Age*		
18-24 years	85	8.6
25-34 years	402	40.6
35-44 years	227	33.9
45-54 years	124	12.5
55-64 years	114	11.5
65-74 years	37	3.7
75+	1	0.1
*Race*		
Asian	87	8.8
Native American	12	1.2
Black	55	5.6
Pacific Islander	2	0.2
White	788	79.6
More than one race	46	4.6
*Hispanic, Latino, or Spanish origin?*		
Yes	87	8.8
No	903	91.2
*Education*		
<HS	4	0.4
HS Grad/GED	93	9.4
Some College	242	24.4
Associate's Degree	111	11.2
Bachelor's Degree	395	39.9
Master's Degree	110	11.1
Professional Degree	25	2.5
Doctorate	10	1.0
*Marital Status*		
Single	401	40.5
Married	483	48.8
Widowed	13	1.3
Divorced	83	8.4
Separated	10	1.0

**Table 2:** Green leafy vegetable and red meat consumption and DHCCBS responses of U.S. men and women.

	Total (n=990)		Men (n=468)		Women (n=522)		
	Median	IQR	Median	IQR	Median	IQR	*p*
Green leafy vegetables (cups per week)	2.25	0.85 - 4.67	2.36	0.83 - 4.76	2.16	0.89 - 4.62	0.743
Red Meat (ounces per week)	12.17	5.75 - 21.54	14.87	7.60 - 24.69	9.53	4.28 - 17.66	<0.001

	Mean	SD	Mean	SD	Mean	SD	*p*

*Susceptibility*							
Please rate your perceived risk for developing colon cancer in your lifetime:	2.11	0.60	2.20	1.60	2.10	0.60	0.023
*Severity*							
Colon cancer can severely decrease my quality of life	4.69	0.75	4.70	0.70	4.70	0.80	0.837
Colon cancer could lead to death	4.72	0.69	4.70	0.70	4.70	0.70	0.402
*Perceived Benefits*							
If I eat less red meat I could decrease my risk of developing colon cancer	3.79	0.97	3.80	1.00	3.80	1.00	0.876
If I eat more green leafy vegetables I could decrease my risk of developing colon cancer	4.17	0.84	4.20	0.80	4.20	0.80	0.579
*Perceived Barriers*							
I don't like the taste of other protein-rich foods	2.11	1.02	2.10	1.00	2.10	1.00	0.901
I don't like the taste of green leafy vegetables	1.93	1.16	2.00	1.10	1.90	1.20	0.052
I can't imagine never eating red meat	3.22	1.53	3.40	1.50	3.10	1.50	0.011
*Cues to Action*							
A healthcare provider has recommended that I eat less red meat	1.65	1.01	1.80	1.10	1.50	0.90	<0.001
A friend or family member has recommended that I eat less red meat	1.83	1.18	2.00	1.20	1.70	1.10	0.003
A healthcare provider has recommended that I eat more green leafy vegetables	2.65	1.45	2.70	1.50	2.60	1.50	0.068
A friend or family member has recommended that I eat more green leafy vegetables	2.72	1.47	2.90	1.50	2.60	1.50	0.001

A friend or family member has recommended that I eat more green leafy vegetables	2.70	1.50	2.90	1.50	2.60	1.50	0.001

**Table 3:** Subjective measures of red meat and green leafy vegetable intake of U.S. men and women relative to barrier question responses regarding diet preferences.

	I can't imagine never eating red meat				
	Strongly Disagree	Disagree	Neither	Agree	Strongly Agree
*Male N (%)*	85 (18.2%)	74 (15.8%)	50 (10.7%)	112 (23.9%)	147 (31.4%)
Mean weekly RM ounces (IQR)	10.24 (0.12-13.04)	15.36 (5.22-18.11)	15.72 (5.68-20.75)	20.28 (12.16-25.04)	24.36 (12.25-31.62)

*Female N (%)*	124 (23.8%)	93 (17.8%)	40 (7.7%)	137 (26.2%)	128 (24.5%)
Mean weekly RM ounces (IQR)	5.80 (0.00-6.40)	9.51 (4.27-12.32)	13.35 (5.24-15.23)	15.21 (7.57-20.58)	21.04 (8.70-28.41)

	I don't like the taste of green leafy vegetables				
	Strongly Disagree	Disagree	Neither	Agree	Strongly Agree

*Male N (%)*	199 (42.5%)	153 (32.7%)	48 (10.3%)	50 (10.7%)	18 (3.8%)
Mean weekly GLV cups (IQR)	4.96 (1.69-6.50)	2.88 (0.92-4.02)	2.30 (0.67-2.95)	2.01 (0.16-1.83)	0.70 (0.00-0.68)

*Female N (%)*	281 (53.8%)	133 (25.5%)	30 (5.7%)	55 (10.5%)	23 (4.4%)
Mean weekly GLV cups (IQR)	4.41 (1.54-5.89)	2.59 (0.61-2.98)	2.78 (0.55-3.53)	1.86 (0.26-2.31)	1.26 (0.05-1.40)

## Data Availability

The data used to support the findings of this study are available from the corresponding author upon request.
